# The Impact of Clinical Characteristics of Patients with Mental Disorders on the Long-Term Effects of Electroconvulsive Therapy

**DOI:** 10.3390/jcm15114285

**Published:** 2026-06-01

**Authors:** Floris Petru Iliuță, Mirela Manea, Aliss-Mădălina Mareș, Corina Ioana Varlam, Florentina Ionela Lincă, Teodor-Georgian Nuță, Mihnea Costin Manea

**Affiliations:** 1 Department of Psychiatry and Psychology, Discipline of Psychiatry, Faculty of Stomatology, “Carol Davila” University of Medicine and Pharmacy, 010221 Bucharest, Romania; floris.iliuta@umfcd.ro (F.P.I.); mirela.manea@umfcd.ro (M.M.); aliss-madalina.mares@drd.umfcd.ro (A.-M.M.); mihnea.manea@umfcd.ro (M.C.M.); 2 Department of Psychiatry, “Prof. Dr. Alexandru Obregia” Clinical Hospital of Psychiatry, 041914 Bucharest, Romania; corina-ioana.varlam@rez.umfcd.ro; 3 Department of Special Psychopedagogy, Faculty of Psychology and Educational Sciences, University of Bucharest, 050663 Bucharest, Romania; 4 Psychiatry Research Laboratory, “Prof. Dr. Alexandru Obregia” Clinical Hospital of Psychiatry, 041914 Bucharest, Romania

**Keywords:** electroconvulsive therapy, psychiatric rehospitalization, long-term outcomes, illness duration, treatment-resistant mental disorders, remission durability, clinical predictors, relapse prevention, neuroplasticity

## Abstract

**Background**: Electroconvulsive therapy (ECT) remains an effective intervention for severe and treatment-resistant psychiatric disorders. While acute response to ECT is well established, clinical predictors of long-term outcomes remain insufficiently characterized. This study examines the impact of illness duration on long-term ECT effectiveness, operationalized specifically as the amount of time elapsed from treatment completion to psychiatric rehospitalization. By controlling for primary diagnosis, psychiatric comorbidities, sleep disorders, and number of ECT sessions, this study aims to clarify the role of illness chronicity in sustaining remission and reducing relapse risk, while recognizing rehospitalization as a primary indicator of long-term clinical stability. **Methods**: We conducted a retrospective cohort study of 249 patients treated with ECT at a tertiary psychiatric hospital. Time to first psychiatric rehospitalization following completion of ECT was used as a proxy for relapse. Illness duration was analyzed alongside primary diagnosis, psychiatric comorbidities, sleep disorders, and number of ECT sessions to assess its independent association with long-term outcomes. **Results**: Nearly half of patients (44.6%) demonstrated sustained clinical benefit, defined by delayed or absent rehospitalization during a three-year follow-up period. Shorter illness duration emerged as a key predictor of more durable remission. Patients with illness duration below 15 years, and particularly those with less than 5 years of illness, experienced significantly longer periods without rehospitalization compared with individuals with longer disease trajectories, despite the latter often receiving more ECT sessions. **Conclusions**: Shorter illness duration is associated with improved long-term outcomes following ECT, suggesting that earlier intervention may enhance treatment durability. These findings support reconsidering ECT earlier in the course of treatment-resistant psychiatric disorders. Prospective studies integrating clinical, cognitive, and biological markers are warranted to improve patient selection and develop personalized ECT strategies.

## 1. Introduction

Electroconvulsive therapy (ECT), the oldest somatic treatment still used in psychiatry, has been employed since the early 1930s and remains one of the most effective interventions for severe psychiatric disorders. The American Psychiatric Association recognizes ECT as a critical treatment option, particularly when other interventions fail or when rapid symptom relief is required in life-threatening situations such as severe depression, mania, schizophrenia (especially with catatonia), or high suicide risk [1]. Its therapeutic effects are believed to involve modulation of neuronal circuits and neurotransmitter systems, enhancement of synaptic plasticity and neurogenesis, and increased expression of neurotrophic factors such as brain-derived neurotrophic factor (BDNF), which plays a key role in mood regulation and cognitive functioning [2,3]. Despite its proven effectiveness and long-term benefits, ECT remains one of the most debated treatments in psychiatry.

Clinical response to ECT varies considerably among patients, prompting extensive research into predictors of treatment effectiveness. The number of sessions required to achieve remission differs widely: acute episodes may respond within six sessions, whereas chronic or treatment-resistant conditions often require six to twelve treatments [1]. Illness course and prior episode burden appear to influence outcomes; patients with multiple previous episodes have demonstrated reduced symptomatic improvement and poorer quality of life, with approximately a 30% lower chance of response after six ECT sessions [4]. Conversely, shorter illness duration and the absence of medication resistance have been associated with more favorable outcomes, suggesting that illness chronicity plays a central role in treatment success [5,6].

Diagnostic classification also contributes to variability in ECT response. Mood disorders, including major depressive disorder and bipolar disorder, often demonstrate robust clinical and molecular responses to ECT, particularly in treatment-resistant cases [7]. In contrast, psychotic disorders such as schizophrenia may show more variable responses, highlighting the importance of diagnostic context when evaluating treatment effectiveness [8]. Moreover, comorbid psychiatric conditions and specific symptom profiles may further modify treatment outcomes, underscoring the complex relationship between diagnosis and ECT efficacy [8].

Sleep disturbances are among the most common comorbidities in individuals with psychiatric disorders and include difficulties with sleep initiation, maintenance, and early awakening. These disturbances significantly impair daily functioning and quality of life and often prompt individuals to seek medical care [9,10,11]. ECT is hypothesized to improve sleep by enhancing neurotransmission, normalizing neuroendocrine functioning, and promoting neuronal growth and synaptic remodeling, leading to improvements in mood, cognition, objective and subjective sleep parameters, sleep regulation and quality [12,13].

Treatment intensity is another important determinant of long-term outcomes. Across studies, patients typically receive an average of 6–10 ECT sessions, although the total number may vary widely [14,15,16]. The required number of sessions differs by diagnosis and illness severity: fewer sessions may be needed for bipolar depression compared with unipolar depression [15], while schizophrenia and bipolar depression may require more intensive treatment courses than schizoaffective disorder, mania, or major depressive disorder [16]. Evidence suggests that a greater number of ECT sessions may reduce relapse risk and improve survival outcomes, even among patients who initially achieve remission and patients receiving nine or more treatments demonstrated significantly lower readmission rates [17,18]. Overall, most studies report treatment courses within the 6–10 session range, although some report fewer than six sessions and others more than ten [19].

Patient characteristics and symptom profile may also influence response trajectories. Certain neuroanatomical features are associated with different outcomes after ECT, while individuals with severe depressive symptoms may respond more rapidly [20,21,22]. Elderly patients with comparable severity may experience slower improvement [23,24]. However, older age has also been associated with better treatment response in some populations [15,25,26]. In schizophrenia, response rates of approximately 50% have been reported regardless of gender, and treatment protocol variations appear to have limited influence on efficacy [15].

Although numerous predictors of ECT response have been investigated, important gaps remain regarding the long-term durability of treatment effects. Most previous studies have relied on subjective outcome measures such as symptom rating scales and questionnaires, which may not fully capture sustained remission in real-world settings. Consequently, the long-term effectiveness of ECT and the predictors of relapse remain areas of ongoing debate.

In this context, the present study examines the impact of illness duration on long-term ECT effectiveness, operationalized specifically as the amount of time elapsed from treatment completion to psychiatric rehospitalization. By controlling for primary diagnosis, psychiatric comorbidities, sleep disorders, and number of ECT sessions, this study aims to clarify the role of illness chronicity in sustaining remission and reducing relapse risk, while recognizing rehospitalization as a primary indicator of long-term clinical stability. Through the use of an objective outcome measure and a large real-world clinical sample, this research seeks to address an important gap in the literature and contribute to a more precise understanding of long-term ECT effectiveness.

## 2. Materials and Methods

### 2.1. Study Design

A retrospective observational study was conducted over a 10-year period, focusing on the psychiatric hospitalizations of 249 patients at the “Prof. Dr. Al. Obregia” Clinical Hospital of Psychiatry of Bucharest, Romania. This is one of the largest psychiatric medical units not only in the country, but also in South-Eastern Europe, with a total of 1229 beds. The hospital offers emergency psychiatric care with inpatient hospitalization, to residents of Bucharest and surrounding areas, serving as a referral center for complex cases throughout Romania. This study represents a secondary analysis of a dataset that has been previously used in another publication with a different research question [27].

This study aims to investigate the impact of illness duration on the outcomes of ECT, operationalized as the time from completion of ECT sessions to rehospitalization, while controlling for primary diagnosis, secondary psychiatric diagnoses (defined as the presence of at least one additional DSM-5/ICD-10 psychiatric comorbidity documented during the index hospitalization, separate from the primary diagnosis), associated sleep disorders, and the number of ECT sessions. We hypothesize that patients with a longer duration of illness (predictor variable) will exhibit a significantly higher risk of rehospitalization (dependent variable) within a shorter period following ECT, even after statistically accounting for the effects of diagnosis, psychiatric comorbidities, sleep disorders, and total ECT exposure. By objectively measuring the interval between treatment and rehospitalization and examining key covariates, our study seeks to provide a more precise understanding of the long-term effectiveness of ECT and address a notable gap in the existing literature.

The study included adult patients (≥18 years at the time of admission) who were admitted between January 2013 and December 2023 and who underwent ECT for various psychiatric disorders diagnosed according to the Diagnostic and Statistical Manual of Mental Disorders, Fifth Edition, including schizophrenia, psychotic disorder, schizoaffective disorder, bipolar disorder, major depressive disorder (single episode), major depressive disorder (recurrent), and obsessive–compulsive disorder. Patients whose ECT sessions were discontinued owing to treatment-emergent adverse effects were excluded from the study sample. Patients for whom full datasets were not available were excluded from the study sample.

The decision to perform ECT was made by attending psychiatrists based on specific, well-defined clinical indications. These predominantly included severe treatment resistance (defined as the failure of at least two sequential, adequate pharmacological trials), or imminent life-threatening emergencies. Specifically, “suicidal ideation” was restricted to cases of severe, acute suicidal behavior or ideation with a high risk of self-harm that showed poor response to standard treatment or required immediate intervention. Similarly, “catatonia” was operationalized as severe catatonic states that necessitated rapid somatic stabilization or had failed an initial high-dose benzodiazepine challenge. While these baseline indications guided clinical selection, they were not introduced into the multivariate rehospitalization model due to high multicollinearity with primary diagnoses and illness duration.

ECT was discontinued upon achieving remission or when symptoms reached a plateau of improvement over two consecutive treatments. Discontinuation also occurred if patients did not respond to the initial four to six sessions or experienced major complications. Post-ECT, clinical and mental state examinations were conducted to assess for any side effects. The efficacy of ECT was determined through clinical psychiatric evaluation.

Patient demographics, psychiatric diagnoses, duration of illness, number of treatment sessions, and medication usage during and after ECT were extracted from medical records.

The study received approval from the Institutional Research Ethics Committee of “Prof. Dr. Alexandru Obregia” Clinical Hospital of Psychiatry (approval no. 138/13.03.2024) and was carried out in accordance with the Declaration of Helsinki. Confidentiality and complete anonymity for the identity of the patients were maintained.

The final sample of 249 patients was selected based on stringent inclusion criteria designed to ensure data homogeneity and minimize confounding variables. All participants were ECT-naive at study entry, receiving the treatment for the first time in their clinical history during the index episode. The three-year longitudinal follow-up focused exclusively on the durability of the initial ECT course; consequently, none of the patients received additional ECT sessions during any subsequent rehospitalizations within the follow-up period. Regarding the primary outcome, rehospitalization was defined strictly as readmission due to the relapse or exacerbation of psychiatric symptoms related to the primary diagnosis, excluding admissions for somatic or social reasons. To maintain the integrity of the clinical outcome data, patients who died, were transferred to long-term chronic care facilities, or were lost to follow-up for administrative reasons were excluded from the final analysis, ensuring that the findings accurately reflect the rate of psychiatric recurrence post-ECT.

ECT was administered using a brief-pulse constant-current device (MECTA Spectrum 5000Q, MECTA Corporation, Tualatin, OR, USA). The treatment protocol followed a standardized clinical procedure: anesthesia was induced with intravenous propofol (1–1.5 mg/kg), and muscle relaxation was achieved with succinylcholine (0.5–1 mg/kg) to minimize musculoskeletal risks. Patients were pre-oxygenated with 100% oxygen via mask ventilation until the return of spontaneous respiration. Electrode placement was primarily bilateral (bitemporal) to maximize clinical efficacy, given the high prevalence of treatment resistance (61.4%) in our cohort. Stimulus dosing was determined using the dose-titration method during the first session to establish the seizure threshold. Subsequent treatments were delivered at 1.5 to 2 times the initial threshold. Seizure adequacy was monitored via two-channel electroencephalogram (EEG) and motor seizure duration, with a minimum requirement of 25 s of generalized seizure activity. The index course consisted of an average of 6 sessions, typically administered three times per week.

### 2.2. Statistical Analysis

Data analysis was performed using R software (version 4.2.2). In a first step, we applied descriptive statistical techniques to outline the sample profile, calculating means, standard deviations, frequencies and percentages for demographic and clinical characteristics. In the analysis of the main variables—rehospitalization and duration of illness—we focused on median values. This methodological choice is justified by the nature of psychiatric disorders, where data may present asymmetric distributions or extreme values; thus, the median provides a more accurate representation of the “typical patient” and of the homogeneity of the group with respect to the mean, which could be distorted by outliers [28].

Given that the variables included in the study are of a categorical nature, we used a multinomial logistic regression model to test the research hypothesis. This model allowed us to evaluate the relationship between the duration of illness (main predictor) and the risk of post-ECT rehospitalization (dependent variable). To isolate the independent effect of illness chronicity and ensure robustness of the results, we statistically controlled for the influence of other major clinical factors (covariates), according to confounding adjustment methodologies [29]. The model was adjusted for primary diagnosis, secondary psychiatric diagnosis, presence of sleep disorders, and category of number of ECT sessions administered. This analytical framework is essential to determine the extent to which the duration of the pathology conditions long-term therapeutic success and maintenance of remission [30].

In addition, statistical analysis was performed using SPSS (version 26.0). Continuous variables were expressed as mean ± standard deviation (SD) or median (range), while categorical variables were presented as absolute frequencies and percentages. The normality of data distribution was assessed using the Shapiro–Wilk test. To evaluate the primary outcome—time to psychiatric rehospitalization—Kaplan–Meier survival analysis was employed. The Log-rank (Mantel–Cox) test was used to compare survival distributions across different primary diagnoses and illness duration categories. To identify independent predictors of rehospitalization, a Cox Proportional Hazards Regression model was constructed, incorporating variables such as diagnosis, age, number of ECT sessions, and comorbidities. Hazard Ratios (HRs) with 95% Confidence Intervals (CIs) were calculated. Statistical significance was defined as a two-tailed *p*-value < 0.05.

## 3. Results

### 3.1. Socio-Demographic and Clinical Data

The clinical and demographic data of our cohort is available in Table 1. The study included a total of 249 patients aged 18–73 (M = 44.86, SD = 14.63), 144 female and 105 male, 216 from urban areas, 33 from rural areas. The most frequent diagnosed disorder was recurrent major depressive disorder amounting to 96 (38.6%) patients in our sample, while 72 (28.9%) were diagnosed with schizophrenia, 45 (18.1%) had a diagnosis of bipolar disorder-depressive episode, 15 (6%) had a diagnosis of schizoaffective disorder, nine (3.6%) of the participants had a diagnosis of psychotic disorder, six (2.4%) had a diagnosis of obsessive–compulsive disorder, three (1.2%) had bipolar disorder–manic episode and another three (1.2%) patients were diagnosed with major depressive disorder (single episode). With regard to associated disorders, 60.2% of the patients had at least one more psychiatric comorbidity and 59% of the participants had associated sleep disorders.

In our sample, the median illness duration was 5–15 years and the median time to rehospitalization was 1–12 months. In addition, 54.2% of the participants had illness duration between 5 and 15 years, which represented the median category for illness duration whilst 22.9% of the participants had illness duration of less than 5 years and 22.9% had an illness duration of more than 15 years. Regarding the indications for ECT, 153 (61.4%) of the participants had treatment resistance, 54 (27.1%) had suicidal indication, 42 (16.9%) catatonia.

The median number of ECT sessions was six. Most patients had between four and six sessions (105, 42.2%) and 99 of the participants (39.8%) had between seven and twelve sessions. Side effects of ECT, but which did not lead to treatment interruption, occurred in 31.3% of the participants. After ECT, 44.6% of participants were not rehospitalized during the 3-year follow-up period, 48.2% were rehospitalized within 1–12 months, and 7.2% were rehospitalized between 13 and 36 months.

**Figure 1:**
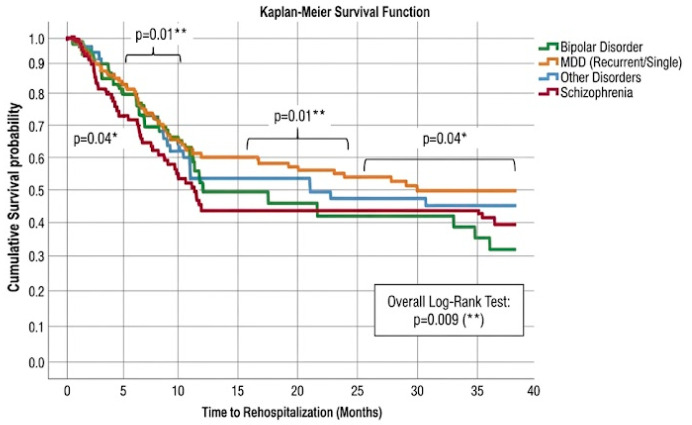
The Kaplan–Meier survival curves. Note: * *p* < 0.05; ** *p* < 0.01.

The clinical outcome analysis reveals that the major incidence of rehospitalizations (48.2%) is heavily concentrated within the first 12 months following the completion of ECT, identifying a critical window of biological and psychopathological vulnerability in the immediate post-treatment period. As demonstrated by the Kaplan–Meier survival curves (Figure 1), a sharp decline in the probability of maintaining remission is observed during the first year across all analyzed diagnostic groups. This early relapse trend reflects the natural clinical progression of severe psychiatric disorders and cannot be attributed to external situational factors, given that readmissions were strictly motivated by the exacerbation of primary psychiatric symptoms.

The severity of our cohort’s clinical profile is further underscored by the fact that 61.4% of patients met treatment-resistance criteria prior to the index ECT intervention. In this context, the high rate of early relapse indicates a fragility of remission that persists despite rigorous maintenance pharmacotherapy. The heterogeneity of long-term therapeutic response is detailed through the analysis of relapse rates stratified by diagnostic categories (Table 2), which confirms that maintaining clinical stability is significantly conditioned by the architecture of the primary pathology. Consequently, patients diagnosed with schizophrenia recorded the highest overall recurrence rate over the 3-year period (59.7%), followed by those with bipolar disorder (54.1%), while the major depressive disorder (MDD) group exhibited a more stable trajectory, with a recurrence rate of 49.5%.

In contrast to the dynamics of the first year, the sporadic distribution of rehospitalization episodes in the 13–36 month interval—representing only 7.2% of the total sample—suggests that patients who manage to maintain stability beyond the critical 12-month post-ECT threshold have a higher statistical probability of preserving longer-term remission. This observation suggests that Kaplan–Meier survival analysis may serve as a useful clinical guide during the informed consent process, rather than an absolute prognostic tool, given the inherent diagnostic and clinical heterogeneity of these patients. By correlating illness duration with specific diagnosis, clinicians can formulate tentative prognostic expectations, providing patients with a realistic perspective on the probability of maintaining remission, while remaining cognizant of individual variations in post-hospitalization maintenance strategies and pharmacological adherence.

On the other hand, detailed analysis of the subgroup of 18 patients who experienced delayed rehospitalizations, representing 7.2% of the total sample, provides preliminary clinical insights into the factors favoring long-term therapeutic stability. While the majority of the cohort (48.2%) relapsed within the first year, this specific group demonstrated remarkable resilience, characterized by superior clinical indicators. Diagnostically, 66.7% of these patients (n = 12) were diagnosed with Major Depressive Disorder, supporting the trend that this pathology follows a more stable post-ECT trajectory in our cohort compared to Schizophrenia or Bipolar Disorder. Furthermore, the chronicity profile played an essential role, with 77.7% of these subjects (n = 14) having an illness duration of less than 15 years. This aligns with our baseline hypothesis that a shorter illness duration is significantly associated with sustained remission (*p* < 0.001), though the 15-year threshold should be interpreted by clinicians as a general proxy for advanced clinical chronicity and cumulative treatment resistance, rather than a rigid, deterministic cut-off point.

Over the entire 3-year follow-up period, the cumulative rehospitalization rates varied across diagnostic categories. Specifically, Other Disorders exhibited the highest overall recurrence rate at 66.6% (n = 20), followed closely by Schizophrenia at 59.7% (n = 43), and Bipolar Disorder at 54.1% (n = 26). Conversely, Major Depressive Disorder demonstrated the lowest cumulative recurrence rate at 49.5% (n = 49). While the differences within the late 13–36 month intervals did not reach statistical significance due to the small remaining sample size (n = 18), the global Log-rank analysis confirms a distinct diagnostic baseline trajectory across the total cohort.

Long-term efficacy was also supported by the intensity of the initial intervention, as all 18 patients completed a full index course of ECT, and 83.3% of them (n = 15) benefited from an optimal number of sessions, ranging between 7 and 12. This correlation suggests that a robust therapeutic regimen in the acute phase can significantly delay the onset of the first relapse beyond the critical one-year threshold. Moreover, the impact of comorbidities was minimal in this subgroup, with only 22.2% (n = 4) presenting sleep disorders, in contrast to the overall prevalence of 59% observed across the entire sample. This finding highlights that the absence of sleep disturbances and multiple psychiatric comorbidities constitutes an essential protective factor for maintaining ECT-induced neuroplasticity. Therefore, patients who succeed in surpassing the first year of stability exhibit an increased probability of deep neurobiological stabilization, offering a model of therapeutic success that can guide prognostic expectations during the informed consent process (Table 3).

### 3.2. Hypothesis Testing

It is assumed that patients with a longer duration of illness (predictor variable) have a significantly higher risk of rehospitalization (dependent variable) within a shorter time frame after completing ECT sessions, even when statistically controlling for the influence of the main diagnosis, psychiatric comorbidities, sleep disorders, and the total number of ECT sessions performed. To test this hypothesis, a multinomial logistic regression model was applied. The Chi-square test indicates a fit of the model to the collected data, χ^2^ (18) = 142, *p* < 0.001 (Table 4).

To ensure the statistical validity and reliability of the multivariate analysis, Table 4 presents the standard model fit measures for the multinomial logistic regression. Although the model evaluates a selective number of clinical predictors, assessing model fit is methodologically necessary to confirm that the final regression structure accurately reflects the observed data without mathematical overfitting. Specifically, the significant model Chi-square (*p* < 0.05) demonstrates that the combination of primary diagnosis, illness duration, and clinical symptoms predicts the timing of rehospitalization significantly better than a baseline, random-chance model. Furthermore, the Pseudo R-squared indices (Cox and Snell, Nagelkerke) provide a standardized estimate of the effect size, indicating the proportion of the variance in rehospitalization risk that is accounted for by our clinical predictors, thereby establishing the mathematical groundwork for the prognostic trends discussed.

The statistical analysis of the data presented in Table 5 reveals a significant correlation between the predictive variables and the risk of rehospitalization, providing a nuanced empirical validation of the stated hypothesis. The results of the multinomial regression model indicate that the duration of illness exerts a critical predictive influence on post-therapeutic stability, even when strictly controlling for confounding variables such as primary diagnosis, psychiatric comorbidities, and the total number of electroconvulsive therapy (ECT) sessions.

Regarding the probability of avoiding rehospitalization altogether (I. No Rehospitalization), the data show a clear advantage for patients with a duration of illness under 5 years (B = 1.503; SE = 777; Z = 1.726; *p* = 0.042; OR = 4.50), compared to the reference group (5–15 years). Although the group with a duration exceeding 15 years also shows an increased probability of remaining out of the hospital in the initial stage (B = 1.314; SE = 532; Z = 1.503; *p* = 0.047; OR = 3.72), this stability appears to erode rapidly over time.

The primary validation of the hypothesis occurs in the analysis of specific timeframes for rehospitalization. Patients with a duration of illness exceeding 15 years present a risk of rehospitalization more than nine times higher in the 13–24 month interval (B = 73.622; SE = 1.038; Z = 71.243; *p* < 0.001; OR = 9.43) and nearly seven times higher in the 25–36 month interval (B = 68.715; SE = 10.668; Z = 6.490; *p* < 0.001; OR = 6.92), relative to the medium-duration reference group. These values emphasize that as the illness becomes chronic, clinical resilience decreases significantly, leading to a relapse rate that necessitates hospitalization within a relatively short post-intervention window.

From the perspective of control variables, the model highlights the massive impact of the primary diagnosis and adherence to the treatment protocol. The primary diagnosis proves to be the major determinant for the absence of rehospitalization (B = 7.173; SE = 2.111; Z = 9.561; *p* = 0.018; OR = 1303.73), while the ECT session category functions as a robust protective factor, significantly increasing the odds of non-rehospitalization (B = 5.483; SE = 1.338; Z = 6.428; *p* = 0.013; OR = 240.56). However, the presence of comorbidities, particularly sleep disorders (B = 3.794; *p* = 0.026) and secondary diagnoses (B = 1.288; *p* = 0.030), introduces vectors of instability that may precipitate a return to the clinical setting.

Particular attention must be paid to the 95% Confidence Intervals (CIs) presented in the table, which provide a measure of the precision of the estimates and the reliability of the results. In the case of variables with an extreme impact, such as the primary diagnosis in the first section (CI: 20.78–819,23.63) or the session category (CI: 17.40–3326.04), we observe very wide intervals. This range suggests high inter-individual variability in treatment response, although the lower bound remains above one, confirming the statistical significance of the effect. Conversely, for rehospitalization in the 13–24 month interval, the confidence intervals for duration of illness > 15 years (CI: 7.23–11.13) and for sleep disorders (CI: 4.12–7.92) are much narrower and more precise. This high precision indicates a consistency of risk across the studied population, strengthening the certainty that long-term duration of illness and the presence of comorbidities are constant and robust predictors of medium-term rehospitalization.

In conclusion, the results confirm that a duration of illness exceeding 15 years constitutes a major vulnerability factor for rehospitalization. Although ECT intervention provides a substantial therapeutic benefit, the capacity to maintain this state in the medium and long term is inversely proportional to the longevity of the pathology. Thus, the hypothesis is confirmed: chronic patients present a significantly higher risk of rehospitalization within a shorter timeframe, a phenomenon that persists regardless of the complexity of the diagnosis or the intensity of the treatment administered.

## 4. Discussion

Chronic mental illness is associated with substantial disability, and despite adequate pharmacological treatment, a considerable proportion of patients fail to achieve sustained symptom remission, prompting consideration of electroconvulsive therapy (ECT) as a therapeutic option. The present study contributes to this field by examining clinical predictors of long-term ECT effectiveness, using time to rehospitalization as an objective real-world outcome.

ECT remains an established intervention for patients with inadequate treatment response and those presenting with severe acute symptoms, including psychosis, catatonia, or persistent suicidal ideation, in line with recommendations from major psychiatric associations [1]. Consistent with these indications, 61.4% of participants in our cohort exhibited treatment resistance, 27.1% presented with suicidal ideation, and 16.9% had catatonia at the time of ECT initiation.

Previous studies and meta-analyses have consistently demonstrated the efficacy of ECT across a range of psychiatric disorders and have identified several predictors of ECT response. Notably, clinical factors, including baseline severity, older age, presence of psychotic features, and treatment resistance, significantly influence both response and remission rates after ECT in major depression [31]. Additionally, shorter illness duration and shorter duration of the current episode have emerged as robust predictors of improved outcomes [14,15,16,32]. Our findings extend this literature by demonstrating that illness duration also predicts longer-term outcomes following ECT, with patients with shorter illness trajectories exhibiting more durable remission, as reflected by delayed or absent rehospitalization.

Reported response rates to ECT vary considerably depending on diagnosis, symptom severity, and methodological differences across studies. Chanpattana et al. [15], Pawelczyk et al. [16], and Kaster et al. [19] reported response rates of 54.5%, 60.0%, and 76.7%, respectively. However, there remains no consensus on how ECT response should be defined, with most studies relying on symptom-rating scales [19,33]. In contrast, our study employed time to rehospitalization as a pragmatic indicator of sustained clinical benefit. Using this approach, 44.6% of patients were not rehospitalized during the three-year follow-up, and an additional 3.6% were rehospitalized only after 25 months, yielding an overall response rate of 48.2%, which aligns with estimates reported in literature.

Psychiatric comorbidities have been associated with reduced ECT response in prior studies [8,16,34,35]. To minimize bias, we incorporated primary diagnosis, secondary psychiatric diagnoses, sleep disorders, and number of ECT sessions as covariates in our regression model. This strategy allowed us to isolate the specific contribution of illness duration while controlling for key clinical factors, thereby strengthening the validity of our findings.

Consistent with existing recommendations, the median number of ECT sessions in our cohort was six, falling within the commonly reported therapeutic range of 6–10 sessions [5,7,34]. This suggests that treatment delivery in our sample closely reflected standard clinical practice.

Large cohort studies have reported reduced early rehospitalization rates among patients receiving ECT. Slade et al. observed up to a 46% reduction in 30-day readmissions among patients with severe affective disorders [36], while another paper similarly reported lower risks of early and frequent readmissions [37]. Nevertheless, longer-term protection appears more variable. Some studies have found no significant reduction in rehospitalization following ECT [38], and relapse remains common, with approximately 40% of patients rehospitalized within six months and over half within one year [39]. These figures are consistent with more recent follow-up studies of mixed affective disorder cohorts reporting six-month relapse rates of 37–50% [36,40,41]. Socio-demographic predictors of ECT response have generally shown limited influence [36,42].

In contrast to these findings, our results indicate that illness duration is a clinically meaningful predictor of medium to long-term outcomes following ECT. Patients with longer illness trajectories exhibited the highest rehospitalization risk between 13 and 24 months, with persistently elevated risk extending to 25–36 months. These findings suggest that while ECT provides substantial short-term benefits, these effects may be less durable in individuals with longstanding illness. From a clinical perspective, this supports earlier consideration of ECT in treatment-resistant patients, rather than reserving it as a late-stage intervention only.

When examining the impact of illness chronicity, our study utilized specific duration thresholds (<5 years, 5–15 years, and >15 years) established a priori based on standard psychiatric frameworks of disease progression. As detailed in major reference literature, such as Kaplan & Sadock’s Comprehensive Textbook of Psychiatry, these intervals reflect distinct clinical and neurobiological phases. While a duration of less than 5 years represents an early to intermediate window with typically higher treatment responsiveness, a duration exceeding 15 years serves as a well-recognized operational marker for severe, ultra-chronic illness. At this advanced stage, cumulative neuroprogressive changes and long-standing treatment resistance heavily predispose patients to highly unstable remissions and a heightened vulnerability to rapid relapse.

However, these statistical cut-off points must be interpreted with caution in real-world clinical practice. Although our multinomial logistic regression model confirmed that a disease duration of over 15 years represents an independent risk factor for accelerated rehospitalization, this finding should not be viewed as a rigid, mechanical rule. Clinicians should treat the 15-year threshold as a general proxy for advanced clinical chronicity and increased relapse risk, rather than an absolute contraindication or a criterion for restricting patient eligibility for Electroconvulsive Therapy.

The novelty of the present study lies in its focus on long-term outcomes using an objective measure (time to rehospitalization) rather than symptom rating scales alone. Additionally, we evaluated illness duration under rigorous control of diagnosis, psychiatric comorbidities, sleep disorders, and treatment intensity, providing a more precise estimate of its independent contribution to sustained remission. To our knowledge, few prior studies have systematically examined extended post-ECT outcomes using this approach.

The findings of the present study should be interpreted in light of several methodological limitations. First, its retrospective cohort design and reliance on data from a single tertiary psychiatric hospital may limit the external validity of our conclusions. Because the data were collected from a tertiary referral center, the cohort naturally represents a highly complex clinical population with a greater degree of symptom severity and treatment resistance compared to patients managed in community or secondary care settings. This concentration of refractory cases may inherently influence the observed rehospitalization rates and the perceived durability of the ECT effect. Therefore, caution should be exercised when generalizing these results to broader, less acute patient populations, and future multi-center studies across diverse healthcare settings are warranted to validate our findings.

Second, a significant confounding factor in this 10-year chart review is the inability to fully control for critical outpatient clinical and psychosocial variables. Although we accounted for baseline clinical demographics, our historical medical records lacked granular, standardized data regarding post-discharge medication categories, precise dosages, financial or structural medication availability, and objective medical compliance. In real-world psychiatric practice, post-ECT maintenance pharmacotherapy regimens are highly heterogeneous and heavily modulate long-term stability. Consequently, the observed relationship between advanced illness duration and accelerated rehospitalization may be partially mediated by underlying medication resistance or variations in post-discharge compliance. Furthermore, vital psychosocial buffers—such as the strength of the patient’s family support system, and qualitative parameters including patient or caregiver feedback, attitudes, and stigma regarding ECT—could not be systematically tracked, thereby representing a constraint that future prospective research must address.

Third, our operationalization of treatment outcome was restricted exclusively to the time to the first psychiatric rehospitalization as a proxy for initial severe relapse. While hospital readmission serves as a robust and objective marker, it fails to capture the full spectrum of long-term ECT outcomes, such as outpatient symptomatic fluctuations, cognitive side effects, social functioning, functional recovery, or global quality of life. Our findings must therefore be interpreted strictly within the context of readmission risk rather than overall clinical status.

Finally, because our statistical survival model focused solely on the time to the first event, the specific clinical reasons, patterns, and frequencies of repeated, multiple rehospitalizations (multi-recidivism) following the first baseline relapse were not evaluated. Longitudinal variations in clinical severity, subsequent non-compliance, or accumulating psychosocial stressors driving multiple readmissions fell outside the primary scope of this study. Prospective studies incorporating standardized symptom scales, comprehensive cognitive assessments, and longitudinal biological measures are essential to validate, refine, and extend the clinical insights presented here.

Recent molecular evidence suggests that ECT induces broad neuroplastic changes through modulation of neurotransmitter systems, neurotrophic factors such as BDNF, inflammatory pathways, and neuroendocrine regulation [43,44]. These large scale biological changes may partially explain why earlier intervention in the illness course is associated with more durable therapeutic response, whereas prolonged illness may reflect more entrenched neurobiological alterations [3,35,45,46,47,48]. ECT has also been shown to influence the hypothalamic–pituitary–adrenal axis, normalize cortisol secretion, and reduce neuroinflammation, processes that may contribute to symptom improvement and relapse prevention [2,3,45,49]. Furthermore, ECT appears to promote neurogenesis and synaptic remodeling, suggesting that adequate treatment exposure may be required to achieve sustained neurobiological recovery [45,50].

Our findings further support growing efforts to identify clinical predictors of ECT response to enable more personalized treatment strategies. While the molecular mechanisms of ECT remain incompletely understood, accumulating evidence indicates that it triggers a cascade of cellular and genetic changes related to synaptic functioning, neuroplasticity, and apoptosis [2,45,51]. Future studies integrating clinical trajectories with molecular and inflammatory markers may help refine patient selection and optimize ECT protocols.

## 5. Conclusions

In conclusion, from a clinical perspective, our findings provide a real-world confirmation of early-intervention theories in treatment-resistant psychiatric populations, rather than aiming to present a fundamentally novel paradigm. The study statistically validates the association between a disease duration of over 15 years and an accelerated risk for rapid rehospitalization. Specifically, the odds for these patients to return to the hospital are more than nine times higher in the 13–24 month interval (OR = 9.43; *p* < 0.001) and nearly seven times higher in the 25–36 month interval (OR = 6.92; *p* < 0.001). Although acute ECT courses and an accurate primary diagnosis provide robust initial stability, the capacity to maintain long-term remission appears inversely proportional to the longevity of the pathology—a phenomenon that persists independently of the number of sessions performed or the presence of comorbidities.

The narrow confidence intervals for these chronic patients (CI: 7.23–11.13 for the 13–24 month interval) indicate that this vulnerability represents a consistent clinical trend within our cohort rather than a random variation. Rather than reserving Electroconvulsive Therapy strictly as a final, late-stage intervention when advanced chronicity is deeply established, these data support the clinical rationale for considering ECT earlier in the therapeutic trajectory of treatment-resistant patients to potentially optimize long-term stability. Ultimately, this research emphasizes the necessity of personalized, post-hospitalization monitoring strategies for patients with severe illness chronicity, as their risk profile remains high even following acute therapeutic success with ECT, being significantly influenced by factors such as sleep disorders and the complexity of secondary diagnoses.

## Figures and Tables

**Table 1:** Clinical and demographic characteristics of participants (N = 249).

Variable	N/Value	%
Age		
Mean (SD)	44.86 (14.63)	
Range	18–73 years	
Residence		
Urban	216	86.7
Rural	33	13.3
Gender		
Male	105	42.2
Female	144	57.8
Primary Diagnosis		
Schizophrenia	72	28.9
Psychotic disorder	9	3.6
Schizoaffective disorder	15	6.0
BD—depressive episode	45	18.1
BD—manic episode	3	1.2
MDD—single episode	3	1.2
MDD—recurrent	96	38.6
OCD	6	2.4
Indication for ECT		
Treatment resistance	153	61.4
Suicidal ideation	54	21.7
Catatonia	42	16.9
Number of ECT Sessions		
1–3	36	14.5
4–6	105	42.2
7–12	99	39.8
>13	9	3.6
Psychiatric Comorbidities		
No	99	39.8
Yes	150	60.2
Sleep Disorders		
No	102	41.0
Yes	147	59.0
Duration of Illness		
<5 years	57	22.9
5–15 years	135	54.2
>15 years	57	22.9
Side Effects of ECT		
No	171	68.7
Yes	78	31.3
Rehospitalization (3-year follow-up)		
Not rehospitalized	111	44.6
1–12 months	120	48.2
13–24 months	9	3.6
25–36 months	9	3.6

Abbreviations: SD = standard deviation; BD = bipolar disorder; MDD = major depressive disorder; OCD = obsessive–compulsive disorder; ECT = electroconvulsive therapy.

**Table 2:** Detailed recurrence and rehospitalization rates by primary diagnosis (3-year follow-up).

Primary Diagnosis	Total Patients (N)	Rehospitalized (N)	Recurrence Rate (%)
Schizophrenia	72	43	59.7%
Bipolar Disorder	48	26	54.1%
Major Depressive Disorder	99	49	49.5%
Other Disorders	30	20	66.6%

**Table 3:** Comparative clinical profile: late rehospitalization subgroup vs. total cohort.

Variable	Late Rehospitalization Subgroup (n = 18)	Total Cohort (N = 249)	Clinical Significance
Primary Diagnosis (MDD)	66.7% (n = 12)	39.8% (n = 99)	MDD shows higher long-term stability
Illness Duration < 15 years	77.7% (n = 14)	22.9% (n = 57)	Shorter duration predicts late/no relapse
ECT Sessions (7–12 sessions)	83.3% (n = 15)	39.8% (n = 99)	Robust index course delays recurrence
Absence of Sleep Disorders	77.8% (n = 14)	41.0% (n = 102)	Sleep quality acts as a protective factor
Treatment Resistance (Pre-ECT)	100% (n = 18)	61.4% (n = 153)	ECT is effective even in resistant cases

Note: Sleep disorders were defined strictly as documented clinical diagnoses of insomnia or sleep-wake disruptions according to ICD-10/DSM-5 criteria during the index admission. Treatment-resistant cases were defined prior to ECT initiation based on the failure of at least two sequential adequate trials of different pharmacological classes.

**Table 4:** Model fit measures.

Model	Deviance	AIC	BIC	McFadden R^2^	χ^2^	df	*p*-Value
1	332	374	448	0.299	142	18	<0.001

**Table 5:** Model coefficients rehospitalization.

Outcome and Predictor	Estimate (B)	SE	Z	*p*-Value	Odds Ratio (OR)	95% CI (Lower–Upper)
I. NO REHOSPITALIZATION (Ref: 1–12 months)						
Intercept	1.457	747	1.611	0.041	4.29	[1.99–18.57]
Duration < 5 years (vs. 5–15)	1.503	777	1.726	0.042	4.50	[2.98–20.61]
Duration > 15 years (vs. 5–15)	1.314	532	1.503	0.047	3.72	[1.31–10.56]
Sessions category	5.483	1.338	6.428	0.013	240.56	[17.40–3326.04]
Secondary psychiatric diagnosis	1.288	676	1.826	0.030	3.63	[1.96–13.65]
Sleep disorder	3.794	518	2.531	0.026	44.43	[16.11–122.61]
Primary Diagnostic	7.173	2.111	9.561	0.018	1303.73	[20.78–81,923.63]
---	---	---	---	---	---	---
II. 13–24 MONTHS (Ref: 1–12 months)						
Intercept	31.336	3.342	34.354	<0.001	4.01	[5.81–12.81]
Duration < 5 years (vs. 5–15)	73.950	894	82.378	<0.001	1.23	[0.89–5.53]
Duration > 15 years (vs. 5–15)	73.622	1.038	71.243	<0.001	9.43	[7.23–11.13]
Sessions category	27.953	683	40.903	<0.001	1.31	[1.15–5.31]
Secondary psychiatric diagnosis	2.031	3.383	2.600	0.048	7.62	[5.01–9.54]
Sleep disorder	50.111	1.007	5.008	<0.001	5.72	[4.12–7.92]
Primary Diagnostic	1.667	626	1.066	0.026	5.29	[3.55–12.09]
---	---	---	---	---	---	---
III. 25–36 MONTHS (Ref: 1–12 months)						
Intercept	118.179	21.570	5.479	<0.001	2.35	[1.32–5.59]
Duration < 5 years (vs. 5–15)	79.901	10.902	7.303	<0.001	5.03	[2.25–9.43]
Duration > 15 years (vs. 5–15)	68.715	10.668	6.490	<0.001	6.92	[5.20–8.68]
Sessions category	51.938	4.162	12.479	<0.001	3.62	[1.19–6.26]
Secondary psychiatric diagnosis	64.543	17.408	3.708	<0.001	2.02	[1.61–5.42]
Sleep disorder	17.907	12.091	10.481	0.039	6	[3.31–9.18]
Primary Diagnostic	22.453	14.770	32.101	0.019	5.61	[3.60–8.92]

## Data Availability

The original contributions presented in this study are included in the article. Some data are not publicly available due to ethical and privacy restrictions, as they contain sensitive clinical information of patients who underwent ECT, but are available on reasonable request from the corresponding authors.

## Abbreviations

The following abbreviations are used in this manuscript:

ECT	Electroconvulsive therapy
BDNF	Brain-derived neurotrophic factor
M	Mean
SD	Standard deviation
BD	Bipolar disorder
MDD	Major depressive disorder
OCD	Obsessive–compulsive disorder
AIC	Akaike Information Criterion
BAIC	Bayesian Akaike Information Criterion
CI	Confidence Intervals
